# Leveraging artificial intelligence to validate traditional biomarkers and drug targets in liver cancer recovery: a mini review

**DOI:** 10.3389/fphar.2025.1697608

**Published:** 2025-10-17

**Authors:** Shengjian Wu, Xiaoqiao Chen, Yuxiu Ji, Chi Zhang, Yujie Xie, Bin Liang

**Affiliations:** ^1^ Department of Rehabilitation, The Affiliated Hospital of Southwest Medical University, Luzhou, Sichuan, China; ^2^ Department of Rehabilitation Medicine, Southwest Medical University, Luzhou, Sichuan, China; ^3^ Rehabilitation Medicine and Engineering Key Laboratory of Luzhou, Luzhou, Sichuan, China

**Keywords:** hepatocellular carcinoma, artificial intelligence, recovery, AFP, PIVKA-II, radiomics

## Abstract

Hepatocellular carcinoma (HCC) remains a leading cause of cancer death, and recovery after therapy is shaped by heterogeneous etiologies, genomes and microenvironments. Targeted and immunotherapy combinations have broadened first-line options; yet durable benefit is uneven, and serum/imaging anchors (AFP, AFP-L3%, PIVKA-II, LI-RADS/mRECIST) incompletely resolve residual disease or functional restoration. In this review we summarise AI-enabled radiology, digital pathology and multi-omic/liquid-biopsy analytics that test and refine traditional biomarkers and drug-target readouts, and appraise translational opportunities in composite surveillance and recovery forecasting. We also discuss enduring challenges—including assay standardisation, spectrum bias, data leakage, domain shift and limited prospective external validation—that temper implementation. By integrating established anchors (AFP/AFP-L3%, PIVKA-II, ALBI, contrast-enhanced hallmarks) with AI-derived signals (radiomics/pathomics, cfDNA methylation) and pathway contexts (VEGF–VEGFR, WNT/β-catenin), emerging strategies align predictions with clinical endpoints, individualise therapy and chart hepatic function. Our synthesis provides an appraisal of AI–traditional integration in liver cancer recovery and outlines pragmatic standards—analytical robustness, transparent reporting and prospective, guideline-conformant evaluation—required for clinical adoption. We hope these insights will aid researchers and clinicians as they implement more effective, individualised monitoring and treatment pathways.

## 1 Introduction

Primary liver cancer—dominated by hepatocellular carcinoma (HCC)—remains a leading cause of cancer mortality worldwide and displays marked etiologic, genomic, and microenvironmental heterogeneity that complicates prognostication and therapeutic decision‐making (Balogh et al., 2016; Llovet et al., 2022a; Rumgay et al., 2022; Llovet et al., 2022b). Clinically used serum markers such as alpha-fetoprotein (AFP) and des-γ-carboxy prothrombin/protein induced by vitamin K absence or antagonist-II (DCP/PIVKA-II), alongside contrast-enhanced imaging hallmarks, form the backbone of surveillance and post-treatment monitoring (European Association for the Study of the Liver, 2024; Singal et al., 2023; Aslam et al., 2024). Yet their performance varies with stage, etiology, and assay choice, and their kinetics differ—AFP/AFP-L3% and PIVKA-II show lead–lag behavior relative to imaging, distinct biological half-lives, and inter-assay variability—so no single marker reliably captures minimal/measurable residual disease, early relapse, or post-therapy functional recovery. Contemporary guidance emphasizes risk-stratified surveillance and careful interpretation of AFP with imaging, reflecting both the utility and limitations of traditional markers. Recent studies suggest PIVKA-II may complement or outperform AFP in selected contexts (for example, in recurrence detection or post-transplant follow-up), but results remain heterogeneous across cohorts (Piratvisuth et al., 2023; Parikh et al., 2023; Beudeker et al., 2023; Marsh et al., 2025), reinforcing the need for rigorous, generalizable validation before routine adoption.

Concurrently, the therapeutic landscape has broadened from multikinase inhibition to anti-angiogenic and immune-checkpoint combinations, with benefits that are clinically meaningful but uneven across molecular subtypes and immune phenotypes (Zhu et al., 2024; Jost‐Brinkmann et al., 2023; Kudo, 2022). VEGF-pathway blockade and tyrosine-kinase inhibitors (e.g., lenvatinib and sorafenib) remain foundational drug classes, and guideline updates now incorporate first-line immunotherapy-based options; however, response heterogeneity and primary resistance—frequently linked to oncogenic signaling such as WNT/β-catenin and to immune-excluded tumor ecosystems—underscore the gap between target biology and patient-level benefit (Testa, 2024; Chen et al., 2022; George and Levine, 2021). This variability motivates biomarker strategies that move beyond single-analyte thresholds toward integrated readouts capable of forecasting individual benefit, relapse risk, and trajectories of hepatic functional recovery after locoregional or systemic therapy.

Artificial intelligence (AI) offers a principled route to strengthen biomarker and target validation for liver cancer recovery by integrating multi-scale evidence—radiology (radiomics), pathology (pathomics), multi-omics, and liquid biopsy—into calibrated, testable predictions. In imaging, handcrafted radiomics and deep learning models have associated pre-treatment and peri-treatment features with microvascular invasion, immunotherapy or TACE response, and postsurgical recurrence; in digital pathology, convolutional and transformer-based systems learned prognostic signatures from routine slides; and in spectroscopy-enhanced workflows, label-free optical fingerprints coupled to neural networks achieved rapid tissue classification (Zhong et al., 2022; Su et al., 2023; Yamashita et al., 2021; Saillard et al., 2020). At the same time, field-level evaluations highlight methodological pitfalls that can inflate performance estimates and hinder translation (e.g., spectrum bias, data leakage, inadequate external validation, and domain shift). To address these risks, consensus frameworks and reporting standards—together with radiomics quality criteria—promote analytical validity, transparent reporting, and prospective, multi-site evaluations that are essential precursors to claims of clinical validity and utility. For this review, we define ‘liver cancer recovery’ on three axes—(a) oncologic remission/relapse risk, (b) viable tumor burden adjudicated by mRECIST/LI-RADS Treatment Response, and (c) hepatic functional restoration (e.g., ALBI trajectory and tolerance for procedures)—evaluated across 0–3, 3–12, and >12-month windows that respectively inform early retreatment/confirmation, surveillance intensity and therapy switching, and late-relapse detection with long-term liver-reserve planning.

## 2 Traditional biomarkers and drug targets in liver cancer recovery

Traditional biomarkers used to assess liver cancer recovery span serum proteins, imaging hallmarks, and pathology-based factors that together inform residual disease risk, treatment response, and trajectories of hepatic function after therapy (Chen et al., 2023; Huang et al., 2023; Xia et al., 2024). Alpha-fetoprotein (AFP) remains the most widely used blood marker, but its standalone sensitivity for surveillance and early recurrence detection is limited; combining AFP with isoform measures (AFP-L3%) and des-γ-carboxy prothrombin/protein induced by vitamin K absence-II (DCP/PIVKA-II) improves discriminative performance and is increasingly embedded in composite algorithms such as GALAD (age, sex, AFP, AFP-L3, DCP) (Table 1). Contemporary guidance emphasizes ultrasound and contrast-enhanced imaging as anchors for monitoring, with arterial-phase hyperenhancement and venous/late-phase washout constituting radiologic hallmarks that support diagnosis and post-treatment assessment (Cannella et al., 2024; Li et al., 2023; Spadarella et al., 2023). In clinical practice, dynamic changes in these markers and imaging features, rather than single thresholds, are interpreted in risk-stratified follow-up pathways.

**TABLE 1:** Core traditional biomarkers and drug-target classes relevant to liver cancer recovery.

Entity	What it measures	Sample/assay	Typical clinical use in recovery context	Key notes
AFP	Oncofetal glycoprotein produced by subsets of HCC	Serum immunoassay	Trend monitoring for recurrence risk; adjunct to imaging in surveillance and post-therapy follow-up	Modest standalone sensitivity; evaluate kinetics and combine with other markers
AFP-L3%	Lens culinaris agglutinin-reactive AFP isoform	Serum lectin fractionation	Complements AFP for early tumor detection and relapse assessment	Interpret as proportion of total AFP; utility greatest when AFP is measurable
DCP/PIVKA-II	Abnormal prothrombin from defective γ-carboxylation	Serum immunoassay	Adjunct for surveillance and early recurrence detection (including post-resection/transplant)	May detect events missed by AFP; assay platforms and cut-points vary
Composite algorithms (e.g., GALAD)	Multivariable score integrating demographics + AFP, AFP-L3, DCP	Calculated from serum markers	Risk stratification for presence/relapse; candidate triage tool alongside imaging	Requires site-specific calibration; performance depends on population mix
Imaging hallmarks	Arterial-phase hyperenhancement with portal/late washout on CT/MRI	Dynamic contrast CT or MRI	Defines viable tumor vs. post-treatment change; informs retreatment timing	Apply standardized acquisition/reading; correlate with serum trends
Pathology: microvascular invasion (MVI)	Tumor emboli in small vessels	Resection/transplant specimen	High relapse risk; guides intensity of post-operative monitoring	Not available after nonsurgical therapy; surrogate imaging/risk models used
Drug targets/classes: VEGF–VEGFR inhibition	Angiogenesis pathway blockade (e.g., bevacizumab; sorafenib/lenvatinib)	Systemic therapy	Foundational backbone; influences necrosis, shrinkage, and perfusion changes on imaging	Benefit modulated by vascular phenotype and liver reserve
Drug targets/classes: immune checkpoint (PD-1/PD-L1) + anti-angiogenic	T-cell activation with vascular normalization	Systemic combination therapy	First-line standard in many settings; impacts durability of response and relapse timing	Efficacy varies with immune-excluded vs. inflamed tumor ecosystems
Oncogenic pathways influencing response (e.g., WNT/β-catenin)	Tumor-intrinsic signaling linked to immune exclusion	Tissue genomics or surrogate signatures	Context for interpreting lack of benefit from immunotherapy	Use as a resistance-context indicator rather than a standalone predictor

Evidence indicates that DCP/PIVKA-II may complement or, in selected settings, outperform AFP for surveillance and recurrence monitoring, including post-resection and post-transplant contexts (Keller et al., 2022; Da-Ano et al., 2020; Jia et al., 2025). Prospective and translational studies show that adding DCP and AFP-L3 to AFP enhances early detection, while several cohorts suggest PIVKA-II tracks recurrence earlier than AFP in a subset of patients; however, effect sizes vary across etiologies and assays, underscoring the need for calibrated cut-points and external validation before universal adoption (Nardone et al., 2024; Zhang et al., 2023; Poetter-Lang et al., 2020; Xu et al., 2023). The GALAD framework has entered late-phase validation, underscoring multivariable models; however, GALAD-type scores should be interpreted alongside imaging trends and marker kinetics (AFP/AFP-L3%/PIVKA-II) rather than as standalone triggers.

Therapeutic targets historically leveraged in hepatocellular carcinoma include the VEGF–VEGFR axis and multi-kinase signaling nodes. Lenvatinib demonstrated non-inferiority to sorafenib in first-line therapy, consolidating VEGFR/FGFR-directed inhibition as a backbone, and the combination of atezolizumab plus bevacizumab improved overall survival *versus* sorafenib, establishing an anti-angiogenic–immunotherapy standard that is now widely adopted (Abou-Alfa et al., 2022; Cheng et al., 2022; Yuan et al., 2023; Qi et al., 2024). Nevertheless, response heterogeneity remains substantial and is partly explained by tumor-intrinsic programs such as WNT/β-catenin (CTNNB1) activation that associate with immune exclusion phenotypes and attenuated benefit from immune checkpoint blockade. These observations justify biomarker strategies that pair traditional serum and imaging readouts with oncogenic-pathway and immune-context indicators when estimating recovery endpoints.

## 3 AI-enabled validation frameworks for biomarkers and targets

AI-enabled validation in liver cancer recovery should proceed as a structured pathway that links analytical validity, clinical validity, and clinical utility while preserving the stated intended use—estimating recurrence risk, anticipating treatment response, and tracking hepatic function restoration (Zhang et al., 2025; Luo et al., 2022; Cao et al., 2025). Prospective protocolization and transparent reporting are essential; early-stage, live clinical evaluations benefit from DECIDE-AI guidance, and studies advancing to randomized or comparative designs should adhere to SPIRIT-AI/CONSORT-AI extensions to minimize bias, clarify integration within clinical pathways, and define decision thresholds and change-management plans.

Analytical validity begins with feature and assay robustness across sites, scanners, and pre-analytics. In imaging pipelines, reproducibility and leakage-avoidant workflows require standardized segmentation, pre-processing, and feature selection with explicit test–retest evidence; widely used checklists (e.g., the radiomics “how-to” and quality tools such as RQS and the newer METRICS score) provide concrete criteria for study design, repeatability checks, model calibration, and external validation (Wang et al., 2021; Liu et al., 2024; Tan et al., 2020). Empirical assessments show that average RQS remains modest across the literature, underscoring the need for prospective registration, phantom/test–retest analyses, and open science artifacts. Harmonization methods are necessary to control batch effects from acquisition or assay variability; ComBat variants and related approaches have demonstrated effectiveness in reducing between-scanner variability of radiomic features, and recent extensions address multi-parameter and covariance shifts seen in multi-centre imaging (Peng et al., 2023; Tejani et al., 2024; Xu et al., 2024; Vasquez-Venegas et al., 2024). Beyond radiomics, similar principles apply to liquid and tissue assays: pre-analytic standardization, cross-platform calibration, and blinded replication should be documented before multi-omic features are combined with clinical variables in risk models.

**FIGURE 1:**
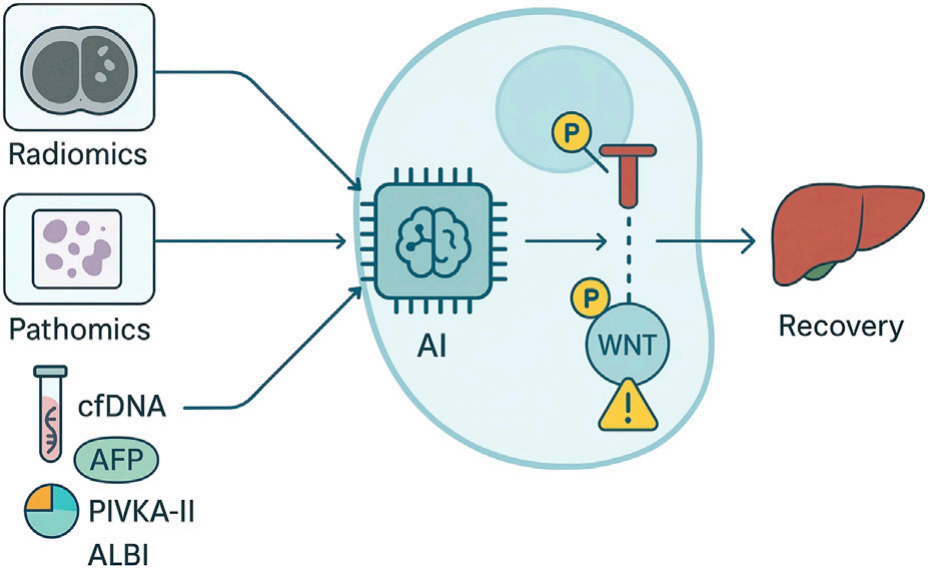
AI-enabled validation of multimodal biomarkers and targets for liver cancer recovery.

Clinical validity requires demonstration that AI-derived readouts generalize across institutions, indications, and sampling frames relevant to routine practice. In HCC, deep learning on whole-slide histology has been externally validated for recurrence risk stratification after resection and can complement conventional pathology factors; AI assistance has also improved pathologist performance in distinguishing primary liver tumor subtypes, highlighting how decision support can interface with expert review rather than replace it (Wang et al., 2024; Soon and Wee, 2020; Vogel et al., 2018). Imaging-based models show that pre-operative CT/MRI radiomics can predict microvascular invasion and relapse risk, but meta-analyses indicate only moderate pooled accuracy to date, emphasizing the need for prespecified cut-points, geography-split validation, and impact analyses before routine use (Vasey et al., 2022; Collins et al., 2021; Whybra et al., 2024). Liquid biopsy adds orthogonal signal: methylation signatures in cfDNA have achieved promising diagnostic performance in HCC and provide a substrate for AI classifiers that may refine surveillance and early-relapse detection when interpreted alongside AFP/PIVKA-II kinetics and imaging trends (Yoshizawa et al., 2025; L et al., 2024; Sakai et al., 2024). As shown in Figure 1, multi-omic integration—combining digital pathology, radiology, circulating biomarkers, and transcriptomic or epigenomic features—can increase discriminative performance and support subtype-aware predictions, but integration must be accompanied by rigorous control of overfitting, transparent feature provenance, and reproducibility across platforms.

Clinical utility requires evidence that AI-augmented decisions improve patient-centred outcomes or operational endpoints without disproportionate harms across subgroups. Early-phase, “silent mode” or decision-support evaluations specified under DECIDE-AI (with clear actionability thresholds and fallback rules) can precede randomized or stepped-wedge deployments registered under SPIRIT-AI/CONSORT-AI.Deployment planning should also include dataset-quality audits, shift/fairness monitoring, and recalibration schedules. Frameworks for medical-AI data quality (e.g., METRIC) distinguish dataset shift (changes in case-mix, scanners, or workflows between development and deployment) from shortcut learning (spurious correlates the model exploits, such as devices or text markers) and from performance disparities across etiologies/geographies; these drive bias-detection, subgroup auditing, and safe model updates under distribution shift (Katsube et al., 2011; Cao et al., 2022; Kocak et al., 2024; Wei et al., 2024). When estimating treatment effect (e.g., benefit from VEGF- or ICI-based regimens), models must be predictive—not merely prognostic—by posing counterfactual questions (individual/conditional average treatment effect) with appropriate adjustment for confounding; otherwise, risk scores reflect baseline prognosis rather than drug-specific benefit.

An AI-enabled validation framework for liver cancer recovery pairs standardized, reproducible analytics with multi-modal external validation and prospective, guideline-conformant evaluation. By enforcing these steps—robust feature engineering and harmonization; transparent modeling with calibration assessed by reliability curves and Brier score; reporting time-dependent decision-curve analysis and net benefit alongside AUC; generalization checks across scanners, assays, and populations; and bias-aware deployment—AI can more credibly forecast recurrence, guide therapy selection, and monitor hepatic function.

## 4 Clinical translation: prognostic/response readouts and recovery monitoring

Clinical translation requires that model outputs align with accepted clinical endpoints and can be acted on within standard pathways for hepatocellular carcinoma. In routine monitoring, dynamic serum markers and standardized imaging response criteria remain the anchors; therefore AI outputs should be actionable endpoints—e.g., ‘probability of viable tumor at next imaging’ and ‘risk of hepatic decompensation within 90 days’—with example threshold ranges (≈30–40% to trigger earlier imaging/loco-regional therapy; ≈10–15% 90-day decompensation risk to avoid TACE/resection) and pre-specified fallback to guideline-concordant management when predictions are indeterminate (Beaufrère et al., 2024; Kocak et al., 2025; Lee et al., 2025). For response assessment, AI can harmonize longitudinal radiology with laboratory kinetics by mapping feature trajectories to categorical readouts used in clinics. mRECIST refinements emphasize viable enhancing tissue as the relevant target, and the LI-RADS Treatment Response algorithm formalizes viability after loco-regional therapy; AI models that predict or emulate these adjudications from serial CT/MRI, together with early on-treatment AFP/PIVKA-II changes, can generate consistent response probabilities and reduce inter-reader variability (Babu et al., 2025; Cabibbo et al., 2025; Lu et al., 2025). Evidence syntheses indicate that delta-radiomics—feature change over time, typically sampled at baseline and first on-treatment imaging (≈6–12 weeks depending on regimen)—improves sensitivity to early therapeutic change; pitfalls include scanner/protocol drift and confounding by treatment-timing; HCC-focused meta-analyses suggest radiomics can predict immunotherapy response, but prospective, multi-centre validation remains limited (Reddy et al., 2022; Olbrich et al., 2024; Zhang et al., 2020), so outputs should include calibrated uncertainty and externally validated thresholds before guiding escalation or de-escalation.

For prognostication, AI adds value by integrating multi-modal signals that capture microscopic vascular dissemination, molecular resistance contexts, and the host–liver axis. Radiomics models for microvascular invasion—an established driver of relapse—show only moderate pooled accuracy in comparative meta-analysis, supporting their use as components of composite risk tools rather than standalone surrogates. Tumor-intrinsic WNT/CTNNB1 activation typifies an ‘immune-excluded’ (non-inflamed) phenotype *versus* ‘inflamed’ tumors; tissue/genomic surrogates (e.g., CTNNB1 mutations, glutamine-synthetase staining or RNA signatures) should be encoded as negative-predictive contexts—modifying probabilities rather than imposing absolute rules—when estimating ICI benefit (Nguyen Hoang et al., 2025; Chen Y. et al., 2024; Fu et al., 2023). In parallel, longitudinal serum kinetics contribute orthogonal information: PIVKA-II has outperformed AFP for early disease in several settings and tracks post-transplant or post-curative recurrence in subsets, enabling AI to weight marker trajectories alongside imaging to forecast near-term relapse risk (Chen L. et al., 2024; Xu et al., 2022; Kim et al., 2023). For recovery monitoring, clinically actionable outputs are continuous estimates of hepatic functional reserve and its trajectory after surgery, loco-regional therapy, or systemic treatment. Albumin–bilirubin (ALBI) grading—computed from serum albumin and bilirubin with grade 1–3 cut-offs—has reproducible prognostic value and, by avoiding subjective ascites/encephalopathy items in Child–Pugh, offers a more objective baseline; AI models that ingest serial labs can project individualized ALBI trajectories and event-risk horizons (Kim et al., 2024; Zha et al., 2025; Bartholomä et al., 2025). Quantitative gadoxetate-enhanced MRI yields indices (e.g., hepatic uptake–based metrics and T1 mapping) that correlate with ALBI and indocyanine-green clearance; fusing these with labs in AI frameworks can forecast post-treatment liver reserve and procedure tolerance for resection, TACE repetition, or systemic-therapy continuation.

Liquid biopsy can further operationalize minimal/measurable residual disease surveillance. Multi-centre data show cell-free DNA methylation assays achieve phase-appropriate performance for detection and surveillance, and emerging prospective studies in HCC indicate that ctDNA status and dynamics stratify molecular residual disease and predict recurrence beyond conventional markers (Wehrle et al., 2024; Ren et al., 2024; Hu et al., 2025; Abdelrahim et al., 2025); embedding these signals with AFP/PIVKA-II kinetics and imaging trends allows AI models to generate calibrated, interval-specific relapse probabilities suitable for risk-stratified follow-up and trial triage.

An implementation-ready translation pathway specifies, in advance, how AI-computed probabilities or risk classes will modify monitoring intensity or therapy selection, demonstrates external validity against mRECIST/LI-RADS and laboratory/imaging standards, and quantifies impact on concrete endpoints such as earlier detection of viable tumor, reduction in unnecessary retreatment, and preservation of liver function. This approach maintains compatibility with guideline-based care while enabling individualized prognostic, response, and recovery readouts that are transparent, reproducible, and auditable.

## 5 Outlook for AI–traditional integration in liver cancer recovery

The near-term priority is to operationalize AI as an adjunct to established serum and imaging anchors by constraining model outputs to clinically accepted targets and by enforcing evaluation standards already outlined for medical AI. Prospective protocols should state the intended use (recurrence forecasting, treatment-response adjudication, hepatic function trajectories) and follow DECIDE-AI/SPIRIT-AI/CONSORT-AI guidance for early “silent-mode” and subsequent impact studies, with explicit decision thresholds and fallback rules (Zhang et al., 2025; Vogel et al., 2018). Analytical validity requires leakage-resistant pipelines, harmonized pre-analytics, and feature/test–retest robustness, supported by radiomics quality criteria and cross-site harmonization strategies before external validation (Wang et al., 2021; Vasquez-Venegas et al., 2024). Calibration of predictions to mRECIST/LI-RADS response and relapse windows is necessary to ensure interoperability with routine reading and scheduling (Beaufrère et al., 2024; Lu et al., 2025).

For prognostication and recovery monitoring, composite tools that integrate AFP/PIVKA-II kinetics, pre-/on-treatment radiology, and tissue or surrogate indicators of oncogenic programs are most likely to generalize. Current radiomics models for microvascular invasion show only moderate pooled accuracy and should be embedded as components of composite scores rather than standalone surrogates, with geography-split validation and prespecified cut-points (Vasey et al., 2022; Zhang et al., 2020). Clinical context modifiers—including WNT/CTNNB1-linked immune exclusion for immunotherapy decision-making, and longitudinal ALBI trajectories for procedure tolerance and liver reserve—should be encoded as negative- or positive-predictive contexts rather than universal rules (Dantzer et al., 2024; Lehrich et al., 2024; Cai et al., 2024). In post-curative and transplant follow-up, weighting of PIVKA-II alongside AFP within multivariable frameworks is reasonable where assay standardization is in place, acknowledging cohort-dependent effect sizes (Keller et al., 2022; Xu et al., 2023; Chen L. et al., 2024).

The most immediate translational gains are expected from multi-modal residual-disease surveillance that fuses cell-free DNA methylation/ctDNA dynamics with calibrated imaging–serology trends to produce interval-specific relapse probabilities suitable for risk-adapted surveillance and trial triage. To sustain performance outside the development domain, deployments should include dataset audits, shift/fairness monitoring, and scheduled recalibration, with subgroup analyses aligned to etiologies and geography. Success metrics should move beyond AUC toward time-dependent net benefit, avoided unnecessary retreatment, earlier detection of viable tumor, and preservation of liver function under standard pathways. If these standards are met, AI will function as a transparent layer that strengthens, and when warranted revises, traditional biomarker and target readouts to individualize surveillance intensity, optimize therapy selection, and forecast hepatic functional recovery within guideline-concordant care.
